# Poster Session II - A312 MATERNAL AND NEONATAL OUTCOMES OF RISANKIZUMAB IN PREGNANT PATIENTS WITH INFLAMMATORY BOWEL DISEASE: A CASE SERIES

**DOI:** 10.1093/jcag/gwaf042.312

**Published:** 2026-02-13

**Authors:** K Li, H Nabavian, S Eisen, K O’ Connor, V Srikanth, J Snelgrove, V W Huang

**Affiliations:** University of Toronto Temerty Faculty of Medicine, Toronto, ON, Canada; University of Toronto Department of Medicine, Toronto, ON, Canada; University of Toronto Temerty Faculty of Medicine, Toronto, ON, Canada; Sinai Health, Toronto, ON, Canada; Sinai Health, Toronto, ON, Canada; Sinai Health, Toronto, ON, Canada; Sinai Health, Toronto, ON, Canada

## Abstract

**Background:**

Biologics are an important therapy for patients with moderate to severe inflammatory bowel disease (IBD). Current guidelines for IBD management during pregnancy recommend continuing biologics, including the newer anti-IL23 agents. Risankizumab (RIS), an anti-IL23 agent, was approved for use for Crohn’s disease (CD) in 2022, becoming available in 2023, and was approved for ulcerative colitis (UC) in 2024. To date, RIS has little evidence regarding use in pregnancy; the WHO pharmacovigilance study including pregnant patients with psoriasis suggested a lower overall frequency of adverse pregnancy outcomes, but a higher reporting rate of abortion and stillbirth compared to TNF-α inhibitors. The PSOLAR study included RIS in the “other biologic” category but reported no RIS specific outcomes. Three pregnancy exposures in IBD patients were reported in the PIANO registry, all of which had term births. Overall, data regarding maternal and neonatal outcomes of RIS are greatly limited in IBD.

**Aims:**

To describe the maternal and neonatal outcomes of five RIS-treated pregnant patients with IBD.

**Methods:**

We conducted a single-centre case series including pregnant patients with IBD treated with RIS at any point during pregnancy seen at the Preconception and Pregnancy in IBD clinic in Toronto between Jan 1, 2017 and Jul 31, 2024. Data was collected on maternal outcomes including live birth, miscarriage, delivery method, and pregnancy-related complications. Neonatal outcomes included preterm birth, small for gestational age (SGA), large for gestational age (LGA), congenital anomalies, jaundice, NICU admission, and APGAR scores.

**Results:**

There were 5 patients (4 CD, 1 UC) treated with RIS during pregnancy. One patient with CD received a dose during first and third trimester only, while others were exposed throughout the entire pregnancy. All delivered healthy, full-term infants. There were 2 (40.0%) vaginal deliveries, 2 (40.0%) elective c-sections, and 1 (20.0%) planned c-section that was done urgently due to evolving pre-eclampsia. One patient experienced a placental hematoma during pregnancy. There were no cases of gestational diabetes, PPROM, IUGR, congenital anomalies, or NICU admissions. One infant was LGA at 96%ile and none were SGA. One infant had jaundice and received bilirubin light phototherapy. Of the 4 available APGAR scores at 1 minute, all were ≥7. At 5 minutes, all 5 infants had APGAR scores ≥7.

**Conclusions:**

In our case series of five RIS exposed pregnancies among patients with IBD, there were no significant adverse maternal or neonatal outcomes. Larger prospective cohort studies are needed to meaningfully assess the safety profile.

**Funding Agencies:**

None

